# Intermediate frequency electrotherapy stimulation to the medial femoris muscle for functional recovery of knee joint after anterior cruciate ligament reconstruction

**DOI:** 10.12669/pjms.38.3.5298

**Published:** 2022

**Authors:** Dejun Song, Yubao Ma, Lihua Zhang, Quansheng Ma

**Affiliations:** 1 Dejun Song, Musculoskeletal Rehabilitation Center, Beijing Rehabilitation Hospital, Capital Medical University, Beijing 100144, P.R. China; 2 Yubao Ma, Musculoskeletal Rehabilitation Center, Beijing Rehabilitation Hospital, Capital Medical University, Beijing 100144, P.R. China; 3 Lihua Zhang, Musculoskeletal Rehabilitation Center, Beijing Rehabilitation Hospital, Capital Medical University, Beijing 100144, P.R. China; 4 Quansheng Ma, Musculoskeletal Rehabilitation Center, Beijing Rehabilitation Hospital, Capital Medical University, Beijing 100144, P.R. China

**Keywords:** Anterior cruciate ligament reconstruction, Electrotherapy, Quadriceps muscle, Knee function

## Abstract

**Objectives:**

To compare the effect of medial femoral muscle stimulation with medium frequency electrotherapy and conventional rehabilitation therapy on knee function recovery after anterior cruciate ligament (ACL) reconstruction.

**Methods:**

Medical records of 50 patients with ACL reconstruction, treated in our hospital between July 2019 and December 2020, were retrospectively analyzed. Patients were divided into control group and study group (n=25, 18 males and 7 females in each group), based on the rehabilitation method used. The control group included patients that received conventional rehabilitation therapy, active quadriceps femoris exercise, traction, and acupuncture. The study group included patients that received medium frequency electrotherapy to stimulate the medial femoris muscle in addition to conventional rehabilitation therapy. The limb circumference recorded before and after the treatment was compared between the two group. The Lysholm scores of the two groups were compared to assess knee function, knee range of motion, and knee motor comfort assessed by visual analogue scale (VAS).

**Results:**

We found similar thigh circumferences, Lysholm scores, knee motion ranges and VAS scores between the patients in both groups before the treatment (P > 0.05). After the treatment, the thigh circumferences and motion ranges were larger, the Lysholm scores higher, and the VAS scores lower in patients of the study group than those in patients of the control group (all *P*s < 0.05).

**Conclusion:**

Intermediate frequency electrotherapy to stimulate the medial femoris muscle can improve knee function and motion range and reduce the patient’s pain after ACL reconstruction.

## INTRODUCTION

The anterior cruciate ligament (ACL) of the knee is frequently injured during sports and military training activities, and this can lead to cartilage and meniscus injury and even to osteoarthritis.^1^,^2^ ACL reconstruction is a common treatment, but it diminishes the quadriceps femoris function causing lower limb muscle atrophy and functional degradation due to the routine leg immobilization brace use required after lower limb surgery. Therefore, rehabilitation exercises to promote the recovery of knee joint function are needed after reconstruction.^3^,^4^

An imbalance of muscle force between the medial oblique and lateral femoris muscles can lead to and can result in lateral patella tracking and pain during knee flexion-extension.^5^,^6^ Transcutaneous electrical muscle stimulation (TEMS) has been used to rehabilitate the lower extremity function after ACL repair/reconstruction.^7^ Medium frequency electrical stimulation of the medial femoral muscle should be useful to improve knee function recovery after ACL reconstruction, but no studies have evaluated this approach. Therefore, we conducted this retrospective study to assess the effect of medium frequency electrical stimulation of the medial femoral muscle on knee function recovery after ACL reconstruction.

## METHODS

Medical records of the patients with ACL reconstruction, treated in our hospital between July 2019 and December 2020, were retrospectively analyzed, a total of 50 patients met the inclusion criteria. Patients were divided into control group and study group (n=25, 18 males and 7 females in each group), based on the therapeutic method used.

### Inclusion criteria

All participants had had a simple cruciate ligament rupture without meniscus, posterior cruciate ligament, or collateral ligament injury and they had undergone a single ACL reconstruction; their age ranged from 18 to 43 years; they all lacked a previous operation history.

### Exclusion criteria

We excluded patients with joint adhesions and vascular nerve injury; patients with heart disease, liver and kidney diseases; and patients with audiovisual and language impairments. According to the above criteria, records of 50 patients with ACL reconstruction in our hospital from July 2019 to December 2020 were selected for the study. The ethics committee of our hospital approved the study (Approval number: 21009, Date: 2021 June 20).

### Therapeutic method

The control group included patients that were treated with conventional rehabilitation therapy sessions including active quadriceps femoris exercise, muscle traction, and acupuncture (30 minutes twice a day, 5 days a week, for 8 weeks).

The study group included patients that received the routine treatment in combination with the medium frequency pulse electrotherapy. After routine treatment, when the patient’s vital signs were stable and the symptoms of nerve defect were not further aggravated, patients were treated with medium frequency pulse electrotherapy. BA2008-III microcomputer bionic therapeutic instrument (Beijing BENAO New Technology Co., Ltd) was used to stimulate the medial femoral muscle. The treatment current of medium frequency electrical stimulation was the medium frequency current modulated by low frequency; The frequency range is set as the intermediate frequency (2000 ~ 4000Hz).^8^ The two output ends are respectively connected with rectangular electrode pieces. The muscle stimulation points are selected at the beginning of medial femoral muscle on the medial patella and 10 cm above the patella, which do not exceed the midline of the thigh. The conversion time between stimulation and rest was set to 15 s; Take the patient’s tolerable tingling, tremor, twitch and muscle contraction as the degree, which shall not exceed 70% of the maximum tolerable intensity with each stimulation lasting between 10 and 20 minutes (4 courses of daily stimulations for 10 days).

### Observation Variables

Medical records of all the patients were analyzed for the following parameters:

The thigh circumference 10 cm above the upper edge of the patella before and after the treatment.
scores to evaluate the knee joint function^9^ before and after treatment (the highest score being 100 for the best function).
Knee ranges of motion measurements before and after treatment.
The larger the range of motion, the better the knee function.
A visual analogue scale (VAS) with 10 consecutive points (0 to 10 points) at equal intervals to allow patients to indicate their perceived knee joint comfort before and after treatment.
The lower the score, the less comfort.

### Statistical Analysis

Data was analyzed using the SPSS 19.0 statistical software. The measurement data expressed in (*x*¯±s). Two independent sample t-test is used for the comparison between the two groups, and paired t-test is used for the comparison before and after treatment in a single group. When *p*< 0.05, it was considered as statistically significant.

## RESULTS

Patients in the control group had a mean age of 32.24 years ± 6.53, a mean body weight of 77.44 kg ± 1.87, a mean height of 175.76 cm ± 2.85. The patients in the study group had a mean age of 32.52 years ±5.8, a mean body weight of 77.48 kg ± 1.71, a mean height of 175.24 cm±. We found no significant differences in the basic data between the two groups (P > 0.05). As shown in Table-I, we found similar circumferences before treatment (P > 0.05). After the treatment, the thigh circumferences of the affected limb in the study group were significantly larger than those in the control group (P < 0.05). We found similar Lysholm scores between the control group and the study group before treatment (P > 0.05). After treatment, the Lysholm scores in the study group were significantly higher than those in the control group (P < 0.05), Table-II.

**Table I:** Comparison of thigh circumference changes in the affected limbs between two groups before and after treatment (*x*¯±s, cm).

Group	n	Before treatment	After treatment	t	P
Control group	25	41.28±0.88	42.30±0.89	4.075	<0.001
Research Group	25	41.30±0.73	44.57±0.77	15.409	<0.001
*t*		0.087	9.644		
*P*		0.931	<0.001		

t and P-values represents the results after independent sample t-test and paired sample t-test, respectively.

**Table II:** Comparison of Lysholm scores between the groups before and after treatment (*x*¯±s).

Group	n	Before treatment	After treatment	t	P
Control group	25	51.27±5.74	61.55±5.36	6.545	<0.001
Research Group	25	51.32±6.04	67.86±6.28	9.491	<0.001
*t*		0.030	3.821		
*P*		0.976	<0.001		

t and P-values represents the results after independent sample t-test and paired sample t-test, respectively.

Motion ranges was also similar before treatment (P > 0.05). After treatment, the motion ranges in the study group were significantly higher than those in the control group (P < 0.05), Table-III VAS scores was also similar before treatment (P > 0.05), Table-IV. After treatment, the VAS scores in the study group were significantly lower than those in the control group (P < 0.05).

**Table III:** Comparison of knee motion ranges before and after treatment between the groups (*x*¯±s).

Group	n	Before treatment	After treatment	t	P
Control group	25	44.39±4.53	57.34±5.73	8.865	<0.001
Research Group	25	44.42±4.62	66.33±6.01	14.451	<0.001
*t*		0.023	5.413		
*P*		0.982	<0.001		

t and P-values represents the results after independent sample t-test and paired sample t-test, respectively.

**Table IV:** Comparison of VAS scores between the groups before and after treatment (*x*¯±s).

Group	n	Before treatment	After treatment	t	P
Control group	25	7.73±1.75	5.39±1.08	5.689	<0.001
Research Group	25	7.89±1.68	4.03±1.02	9.820	<0.001
*t*		0.330	4.577		
*P*		0.743	<0.001		

t and P-values represents the results after independent sample t-test and paired sample t-test, respectively.

## DISCUSSION

The stability of the knee during joint flexion depends on the bone placement, on the restriction of the anterior and posterior cruciate ligaments, and on the balance of the strengths of the quadriceps femoris muscles. After an ACL reconstruction, the biomechanical structure of the lower limb is significantly altered, and the afferent and efferent action potentials of the nerves in the affected limb are significantly reduced, inhibiting the signal transduction and feedback pathway of the knee joint nervous system.^10^,^11^ Moreover, damage to the muscles around the knee can lead to the patients ineffectively maintaining their body balance and the knee injury aggravated.^12^ Correct postoperative nursing and rehabilitation interventions can effectively prevent postoperative complications and promote knee function recovery.^13^ Thus, a rehabilitation plan based on sound scientific and medical technologies based on biodynamics and anatomy can help the healing process and function recovery after knee joint injury and reconstruction.^14^

The biomechanical changes after ACL reconstruction reduce the nerve muscle control and cause quadriceps heads’ asymmetry, which can easily cause secondary knee injuries.^15^ During physiotherapy with electrical stimulation, an electrical current causes muscle contraction, and the current frequency can be modulated to produce low and medium waveforms. The exciting effect on the muscle can enhance the local metabolism, and adaptation is avoided by the application of multiple waves.^16^,^17^ Neuromuscular electrical stimulation on the basis of functional exercise can increase muscle strength and improve limb asymmetry after ACL reconstruction. However, the optimal electrical stimulation frequency varies with the treatment target.^18^ Studies have shown that 2–30 Hz low frequency electrical stimulation may improve the hypoxia tolerance, treadmill fatigue, and swimming exhaustion times in mice; while 10–100 Hz low-frequency electrical stimulation can increase the fibrin content in the gastrocnemius muscle and promote the synthesis of skeletal muscle.^19^,^20^ In our study, a combination of the low-frequency modulated medium frequency electrical stimulation (medium frequency, 2000–4000 Hz and low frequency, 1–167 Hz) was used. This frequency does not stimulate the skin sensory nerve endings and does not cause pain, which makes it ideal to ensure patient tolerance.^21^ Electrical muscle stimulation can improve tension in atrophic muscles and the stability of bones and joints. Under the action of a pulse current, the medial femoral muscle will contract intermittently, promoting the circulation of joint fluid and articular cartilage, and promoting the knee joint recovery.^22^ The technology is well designed and simple and causes no injury or pain, leading to patient compliance.^23^ Our results showed that after treatment, the thigh circumferences, Lysholm scores and knee motion ranges were higher and the pain reduced to a higher degree in the patients that received medium frequency electrotherapy in addition to conventional rehabilitation therapy than in the patients who received conventional rehabilitation therapy alone. Our findings suggest that medial femoris muscle stimulation by medium frequency electrotherapy improves the knee function and motion range of patients after ACL reconstruction.

### Limitations of the study

The main limitations of this study are the small sample size (only 50 patients) and its retrospective nature. Larger, prospective, and retrospective studies, as well as clinical trials are needed to further evaluate the therapeutic effect of medium frequency electrical stimulation in patients after ACL reconstruction.

## CONCLUSION

Medium frequency electrical stimulation of the medial femoral muscle can improve knee function and motion range and reduce the pain of patients after ACL reconstruction.

### Authors’ Contributions:

**DS and QM:** Conceived and designed the study.

**YM & LZ:** Collected the data and performed the analysis.

**DS and QM:** Prepared the manuscript and is responsible for the integrity of the study.

All authors have read and approved the final manuscript.
